# Plant kleptomaniacs: geographical genetic patterns in the amphi-apomictic *Rubus* ser. *Glandulosi* (Rosaceae) reveal complex reticulate evolution of Eurasian brambles

**DOI:** 10.1093/aob/mcae050

**Published:** 2024-03-29

**Authors:** Michal Sochor, Petra Šarhanová, Martin Duchoslav, Michaela Konečná, Michal Hroneš, Bohumil Trávníček

**Affiliations:** Centre of the Region Haná for Biotechnological and Agricultural Research, Crop Research Institute, Šlechtitelů 29, 783 71 Olomouc, Czech Republic; Plant Biosystematics and Ecology Research Group, Department of Botany, Faculty of Science, Palacký University, Šlechtitelů 27, 783 71 Olomouc, Czech Republic; Department of Botany and Zoology, Faculty of Science, Masaryk University, Kotlářská 267/2, 611 37 Brno, Czech Republic; Plant Biosystematics and Ecology Research Group, Department of Botany, Faculty of Science, Palacký University, Šlechtitelů 27, 783 71 Olomouc, Czech Republic; Plant Biosystematics and Ecology Research Group, Department of Botany, Faculty of Science, Palacký University, Šlechtitelů 27, 783 71 Olomouc, Czech Republic; Plant Biosystematics and Ecology Research Group, Department of Botany, Faculty of Science, Palacký University, Šlechtitelů 27, 783 71 Olomouc, Czech Republic; Plant Biosystematics and Ecology Research Group, Department of Botany, Faculty of Science, Palacký University, Šlechtitelů 27, 783 71 Olomouc, Czech Republic

**Keywords:** Apomixis, ddRADseq, geographical parthenogenesis, introgression, private alleles, *Rubus* subgen. *Rubus*

## Abstract

**Background and Aims:**

*Rubus* ser. *Glandulosi* provides a unique model of geographical parthenogenesis on a homoploid (2*n* = 4*x*) level. We aim to characterize evolutionary and phylogeographical patterns in this taxon and shed light on the geographical differentiation of apomicts and sexuals. Ultimately, we aim to evaluate the importance of phylogeography in the formation of geographical parthenogenesis.

**Methods:**

*Rubus* ser. *Glandulosi* was sampled across its Eurasian range together with other co-occurring *Rubus* taxa (587 individuals in total). Double-digest restriction site-associated DNA sequencing (ddRADseq) and modelling of suitable climate were used for evolutionary inferences.

**Key Results:**

Six ancestral species were identified that contributed to the contemporary gene pool of *R.* ser. *Glandulosi*. Sexuals were introgressed from *Rubus dolichocarpus* and *Rubus moschus* in West Asia and from *Rubus ulmifolius* agg., *Rubus canescens* and *Rubus incanescens* in Europe, whereas apomicts were characterized by alleles of *Rubus* subsect. *Rubus*. Gene flow between sexuals and apomicts was also detected, as was occasional hybridization with other taxa.

**Conclusions:**

We hypothesize that sexuals survived the last glacial period in several large southern refugia, whereas apomicts were mostly restricted to southern France, whence they quickly recolonized Central and Western Europe. The secondary contact of sexuals and apomicts was probably the principal factor that established geographical parthenogenesis in *R.* ser. *Glandulosi*. Sexual populations are not impoverished in genetic diversity along their borderline with apomicts, and maladaptive population genetic processes probably did not shape the geographical patterns.

## INTRODUCTION

**Fig. 1.**
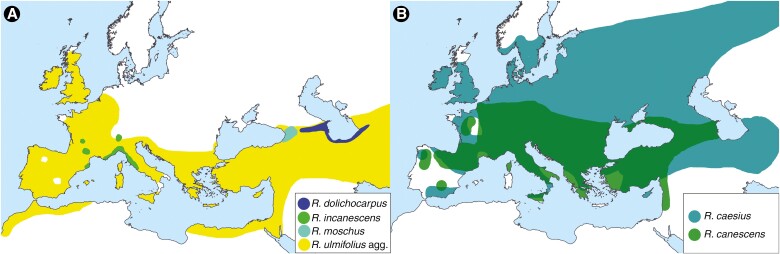
Approximate distributions of *Rubus* ancestors based on the studies by Kurtto *et al.* (2010) and Juzepczuk (1941) and our sampling (Supplementary Data Table S1). *Rubus idaeus* is not included.

**Fig. 2.**
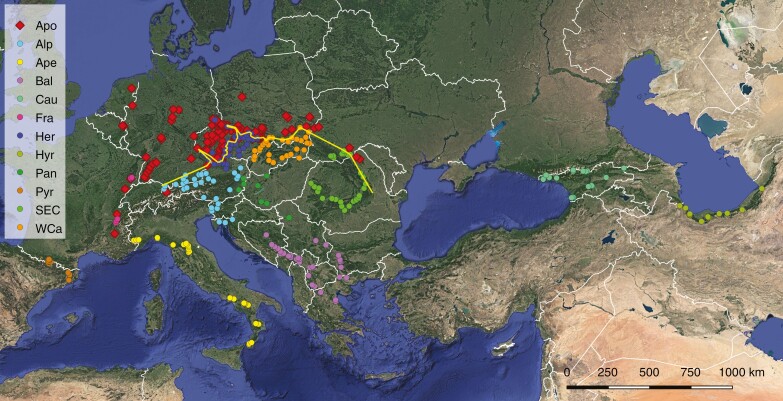
Map of sampled individuals of *Rubus* ser. *Glandulosi*, with their population assignment: apomicts (Apo); sexuals are distinguished according to their geographical origin: Alps (Alp), Apennines (Ape), Balkans (Bal), France (Fra), Hercynia (Her), Pannonia (Pan), Pyrenees (Pyr), Southeastern Carpathians (SEC), Western Carpathians (WCa), Caucasus (Cau) and Hyrcania (Hyr). The borderline between sexuals and apomicts is shown as a yellow line. Background layer: Google Earth.

*Rubus* subgen. *Rubus* (blackberries, brambles) is notorious for its complex evolutionary patterns, the main evolutionary driving forces being hybridization, polyploidization and quaternary range contractions and expansions (Sochor *et al.*, 2015, 2017). Most of the species are polyploid facultative apomicts, i.e. they are able to form seeds both sexually and asexually via gametophytic apomixis (Kurtto *et al.*, 2010; Šarhanová *et al.*, 2012, and references therein). Only five diploid species are extant in Eurasia, namely *Rubus ulmifolius* agg. (including *Rubus ulmifolius* Schott and *Rubus sanctus* Schreb.), *Rubus canescens* DC., *Rubus moschus* Juz., *Rubus dolichocarpus* Juz. and *Rubus incanescens* Bertol. (Gustafsson, 1943; Krahulcová *et al.*, 2013; Kasalkheh *et al.*, 2024). The first two species have a very large distribution, from the Atlantic Ocean to Afghanistan (*R. ulmifolius* agg.) or to Armenia (*R. canescens*; Fig. 1). The former also shows strong inter-regional genetic differentiation, which is traditionally treated taxonomically on the species level: *R. ulmifolius* in the west and *R. sanctus* in the east (Monasterio-Huelin and Weber, 1996). The ranges of the other species are much smaller; *R. moschus* is endemic to the western Lesser Caucasus (Juzepczuk, 1925), *R. dolichocarpus* extends from the central Caucasus to eastern Hyrcania (Kasalkheh *et al.*, 2024), and *R. incanescens* is scattered along the French–Italian Mediterranean coast and some neighbouring areas (Kurtto *et al.*, 2010). Furthermore, two tetraploid sexuals are known from the continent, namely a part of *Rubus* ser. *Glandulosi* (Wimm. et Grab.) Focke and *Rubus caesius* L., both of which have a very large distribution (Figs 1 and 2). Irrespective of the sexual or apomictic reproduction by seeds, all brambles can also spread vigorously by root suckers or rooting primocane tips.

All extant sexuals are known to have contributed in some way to the evolution of polyploid apomicts, with the exception of *R. incanescens*, whose alleles have not yet been detected in any apomict, although it can form triploid hybrids (Sochor *et al.*, 2015). Furthermore, at least one extinct European and one Caucasian sexual have been inferred from phylogenetic studies. Alleles of the extinct European ancestor have been detected particularly in *Rubus* ser. *Nessenses* (whose other parent is *Rubus idaeus* L.) and *Rubus* ser. *Rubus* (i.e. *R.* subsect. *Rubus*, a group formerly called ‘*Suberecti*’), but to a lesser extent also in other series of high-arching brambles. This species was closely related to the erect North American taxa, such as *Rubus* ser. *Canadenses* (L.H. Bailey) H.E. Weber and *Rubus* ser. *Alleghenienses* (L.H. Bailey) H.E. Weber. (Sochor *et al.*, 2015). The second extinct or unknown species with an unsuspected phenotype is presumed to be ancestral for Caucasian polyploids, mainly *R.* ser. *Glandulosi* and *Rubus* ser. *Discolores* (Sochor and Trávníček, 2016).

Forming the opposite extreme of the morphological continuum to *R.* subsect. *Rubus*, tetraploids of *R.* ser. *Glandulosi* are supposed to be the dominant ancestor of prostrate or low-arching brambles characterized by stipitate glands on stems (Sochor *et al.*, 2015). They are likely to have originated from *R. moschus* or its ancestor during the last or preceding interglacial periods. This taxonomically hitherto-unresolved group exhibits a peculiar pattern of geographical parthenogenesis; plants from Northwestern Europe and extra-Carpathian regions of Poland, Ukraine and Romania are facultative apomicts, whereas populations from Southern and Central Europe, the Caucasus and Hyrcania are strictly sexual (Fig. 2). The distribution of genotypic diversity in apomicts implies a secondary contact between the reproductive groups. Consequently, phylogeography and neutral micro-evolutionary processes appear as the main factors propelling geographical parthenogenesis in this taxon (Sochor *et al.*, 2024). However, population genetic processes typical for small fragmented populations (Keller and Waller, 2002; Willi *et al.*, 2013) might also play some role in geographical differentiation between reproductive modes. This concept presumes that marginal populations of sexuals are impoverished in genetic diversity, resulting in reduced adaptability and an inability to compete with apomicts, which are more resistant against genetic drift and inbreeding and outbreeding depression thanks to their fixed heterozygosity (elevated by hybridity and polyploidy) and asexuality (Haag and Ebert, 2004; Tilquin and Kokko, 2016; Sochor *et al.*, 2017). In theory, this hypothesis applies for both primary and secondary contact zones.

Owing to the unique pattern of geographical parthenogenesis and more or less apparent morphological deviations of some apomictic genotypes of *R.* ser. *Glandulosi*, we originally suspected that these apomicts are recently derived from sexual populations either directly or via hybridization with some other (i.e. non-*Glandulosi*; see Table 1) apomictic taxon. Such apomictic taxa, which could potentially pose a source of apomixis for the newly formed *Glandulosi* apomicts, occur in the entire range of sexuals, including the Caucasus and Hyrcania (Kurtto *et al.*, 2010; Sochor and Trávníček, 2016; Kasalkheh *et al.*, 2024). Alternatively, the *Glandulosi* apomicts might already have been formed before the last glaciation, i.e. before the establishment of current geographical and genetic patterns in the European *R.* subgen. *Rubus*. These two scenarios (Pleistocene vs. Holocene origin of the *Glandulosi* apomicts) are expected to result in different patterns of allele sharing between the *Rubus* ancestral taxa and the *Glandulosi* apomicts.

**Table 1.** Taxonomic overview of taxa used in this study, with ploidy levels, reproductive mode, distribution and other notes compiled from Thompson (1997), Kurtto *et al.* (2010), Krahulcová *et al.* (2013), Sochor *et al.* (2015) and van de Beek (2021).

Subgenus	Section	Subsection	Series	Ploidy (2*n*)	Reproduction	Distribution	Notes, prominent species
*Rubus*	*Rubus*	*Rubus*	*Nessenses*	4*x*	Apo^nG^	Europe	Hybridogenous taxa derived from *R. idaeus* and the *Suberecti* ancestor
			*Rubus*	3*x*, 4*x*	Apo^nG^	Europe	Hybridogenous taxa derived predominantly from the *Suberecti* ancestor
			*Alleghenienses*	2x*, 3x*, 4*x*	Sex* + apo^nG^	North America	–
			*Canadenses*	2x*, 3*x*	Sex* + apo^nG^	North America	–
			*Cuneifolii*	2x*, 3x*, 4*x*	Sex* + apo^nG^	North America	–
			*Arguti*	2x*, 3x*, 4*x*, 5x*, 6x*	Sex* + apo^nG^	North America	–
		*Hiemales*	*Canescentes*	2*x*	Sex	Europe, W Asia	Only *R. canescens*^A^
			*Radula*	2*x*, 4x*	Sex + apo*	Europe, W Asia	Sexual diploids *R. incanescens*^A^ and *R. dolichocarpus*^A^
			*Discolores*	(2*x*), 3*x*, 4*x*	(Sex) + apo^nG^	Europe, W Asia	Sexual diploid *R. ulmifolius* agg.^A^
			*Rhamnifolii*	4*x*	Apo^nG^	Europe, W Asia	–
			*Sylvatici*	4*x*	Apo^nG^	Europe, W Asia	–
			*Sprengeliani*	(3x*), 4*x*	Apo	Europe	–
			*Anisacanthi*	4*x*	Apo^nG^	Europe	–
			*Micantes*	4*x*	Apo^nG^	Europe, W Asia	–
			*Vestiti*	4*x*	Apo^nG^	Europe	–
			*Pallidi*	4*x*	Apo^nG^	Europe	–
			*Hystrix*	4*x*	Apo^nG^	Europe	–
			*Glandulosi*	4*x* (5*x*)	Sex^Gs^ + apo^Ga^	Europe, W Asia	Geographical parthenogenesis in tetraploids; most of the tetraploids traditionally treated as ‘*R. hirtus* agg.’; sexual diploid *R. moschus*^A^; apomictic pentaploid *R. nigricans*
	*Caesii*	*–*	*–*	4*x*	Sex	Europe, Asia	Only *R. caesius*^A^
*Idaeobatus*	*Idaeanthi*	*–*	*–*	2*x* (4x*)	Sex	Europe, Asia	In Europe only the diploid *R. idaeus*^A^

*Samples with these properties were not used in this study.

^A^Extant species ancestral for polyploid brambles (sexual ancestors; note that another two ancestors, ‘*Suberecti*’ and an unknown Caucasian species, are probably extinct; see main text).

^Ga^Taxa treated as ‘*Glandulosi* apomicts’ throughout the text.

^Gs^Taxon treated as ‘*Glandulosi* sexuals’ throughout the text and subdivided into 11 populations based on geographical origin.

^nG^Taxa treated as ‘non-*Glandulosi* apomicts’ throughout the text.

However, the phylogenetic and phylogeographical patterns or structuring of genetic diversity have not yet been characterized owing to the lack of polymorphism in the commonly used phylogenetic markers (Sochor *et al.*, 2015) and the absence of high-resolution genomic data in *R.* ser. *Glandulosi*. Taking into consideration all the peculiarities of *R.* ser. *Glandulosi*, we aim to resolve genetic geographical patterns in the group across its range. Specifically, our aims are as follows: (1) to test the role of geographical structure of the genetic diversity of sexuals in the formation of geographical parthenogenesis patterns; (2) to reconstruct the roles of other *Rubus* taxa in the evolution of *R.* ser. *Glandulosi*; (3) to explain the origin of the *Glandulosi* apomicts; and (4) to identify the glacial refugia and postglacial recolonization routes of both sexuals and apomicts of *R.* ser. *Glandulosi*. Ultimately, we evaluate the importance of phylogeography in the formation of geographical parthenogenesis.

## MATERIALS AND METHODS

### Sampling

Owing to the patterns of geographical parthenogenesis, our sampling was focused on tetraploid members of *R.* ser. *Glandulosi* across its distribution range. For this taxon, we sampled individuals analysed in our preceding work (Sochor *et al.*, 2024). Given that complex evolutionary patterns were expected between this taxon and other *Rubus* species, our sampling additionally included other taxa of *R.* sect. *Rubus* and their two known ancestors outside this section, *R. caesius* and *R. idaeus* (Table 1). Owing to the instability of nomenclature and some questionable recent nomenclatural changes, we follow Kurtto *et al.* (2010) for names of taxa and taxonomic concepts. *Rubus* ser. *Glandulosi* (or *Glandulosi* hereafter) samples were divided into 12 groups (hereafter termed ‘populations’) according to their reproductive mode as assessed by the flow-cytometric seeds screen and/or microsatellitegenotyping (Sochor *et al.*, 2024) and their geographical origin (Fig. 2). Given that all apomictic individuals available to us have been genotyped with microsatellites (Sochor *et al.*, 2024), usually one individual per genotype was selected for sequencing. In total, 159 individuals (152 genotypes) were included in the single apomictic population (Apo), covering tetraploids, additionally the common pentaploid *Rubus nigricans* Danthoine (syn. *Rubus pedemontanus* Pinkw.; one genotype with two individuals) and three other pentaploid genotypes from southeastern France. Tetraploid sexual individuals were divided into 11 populations: the Alps (Alp, 31 individuals), Apennines (Ape, 30 individuals), Balkans (Bal, 32 individuals), the French mountains of Chartreuse and Vosges (Fra, six individuals), ynia (Her, 36 individuals), Pannonia (Pan, ten individuals), Pyrenees (Pyr, 17 individuals), Southern and Eastern Carpathians (SEC, 32 individuals), Western Carpathians (WCa, 30 individuals), Caucasus (Cau, 31 individuals) and Hyrcania (Hyr, 24 individuals). Besides the 12 groups from *R.* ser. *Glandulosi*, all extant Eurasian diploid species were included in the study, sampled across their entire ranges wherever possible: *R. ulmifolius* agg. (27 individuals), *R. canescens* (15 individuals), *R. moschus* (eight individuals), *R. dolichocarpus* (25 individuals), *R. incanescens* (four individuals), *R. caesius* (15 individuals), *R. idaeus* (five individuals), and an outgroup of 50 individuals of non-*Glandulosi* apomictic taxa (mostly recognized microspecies) selected to cover most of the other series of *R.* sect. *Rubus* (Table 1). For the complete list of analysed specimens, see Supplementary Data Table S1.

### Whole-genome sequencing and assembly

Owing to the lack of whole-genome reference sequences for the studied group, an *R. moschus* specimen (MS36/14Bs from Adjara, Georgia) was shot-gun sequenced using two approaches. First, long reads were generated by Oxford Nanopore Technologies approach (ONT, Oxford, UK) using Rapid Sequencing kit SQK-RAD004 and the MinION sequencing device and two R9.4.1 flow cells, following the manufacturer’s instructions. The genomic DNA was extracted using Invisorb Spin Plant Mini Kit (Invitec Molecular, Berlin) and subsequently size selected for fragments of >40 kbp by the Short Read Eliminator XL Kit (Circulomics, Baltimore, MD, USA). Basecalling was performed in the software MinKNOW v.21.02.1 using the DNA High-Accuracy algorithm. Second, short high-accuracy reads were generated from the same specimen by Macrogen Europe (Amsterdam, The Netherlands) on the Illumina Novaseq6000 sequencing platform in the 2 × 150 bp configuration, using the TruSeq DNA PCR-Free kit with a 350 bp insert for library preparation.

The ONT data were checked in FastQC v.0.11.9 (Andrews, 2010), and adaptor sequences were trimmed in LongQC v.1.2.0c (Fukasawa *et al.*, 2020). NanoFilt v.2.8.0 (De Coster *et al.*, 2018) was used for filtering data on the minimum read length of 150 bp, minimum average read quality score of five, and ten nucleotides were trimmed from the start of each read. The whole-plastome sequence was assembled from the Illumina data in GetOrganelle v.1.7.6.1 (Jin *et al.*, 2020) using kmer sizes of 21, 45, 65, 85 and 105 and the Embryophyta plant plastome database. Completeness of the sequence was checked visually, in alignment with publicly available *Rubus* plastomes. ONT sequences that mapped on the plastome sequence were subsequently filtered out via mapping in Minimap2 (v.2.24; Li, 2018), and only non-plastome reads were used for *de novo* genome assembly in Smartdenovo (Liu *et al.*, 2021), with default parameters and kmer size set to 19. Subsequently, the sequence was polished in Medaka v.1.6.0 (https://github.com/nanoporetech/medaka) using the base-called ONT reads and in Nextpolish v.1.3.0 (Hu *et al.*, 2020) using the Illumina data. Finally, the contigs were scaffolded into chromosomes in RagTag v.2.1.0 (Alonge *et al.*, 2022) using its scaffold function and two reference genome sequences: *R. ulmifolius* ‘Burbank Thornless’ genome ver. 1 (https://www.dnazoo.org/assemblies/Rubus_ulmifolius) and *Rubus occidentalis* genome ver. 3 (VanBuren *et al.*, 2018). BUSCO analysis (v.5.4.3; Manni *et al.*, 2021) was performed with 2326 BUSCO groups of the Eudicots database. All the computationally demanding calculations were performed on the Czech National Grid Infrastructure MetaCentrum.

### Double-digest restriction site-associated DNA sequencing

Genomic DNA was extracted from silica gel-dried leaves by the cetyltrimethylammonium bromide (CTAB) method (Doyle and Doyle, 1987) and purified by Mag-Bind TotalPure NGS (Omega Bio-tek, Norcross, GA, USA), or using the Invisorb Spin Plant Mini Kit (Invitec Molecular, Berlin, Germany). In both cases, it was finally purified by precipitation with sodium acetate (concentration of 0.3 m in the DNA isolate) and ethanol (100 %, double the DNA volume). The quality of DNA was checked using 1.5 % agarose gel electrophoresis, and the concentration was measured with a Qubit 4 fluorometer with 1X dsDNA HS Assay Kit (Thermo Fisher Scientific, Waltham, MA, USA). As the basis for double-digest restriction site-associated DNA (ddRAD) library protocol development, the protocol of Peterson *et al.* (2012) was followed. First, 100 ng of DNA was cleaved with PstI HF and MseI restrictases in CutSmart buffer (New England Biolabs, Ipswich, MA, USA; 3 h at 37 °C). P1 and P2 barcoded adapters corresponding to the restriction site of the enzymes were ligated immediately afterwards with T4 ligase (New England Biolabs) at 16 °C overnight. The enzymes were heat killed with 65 °C for 10 min. All samples with the same P2 and different P1 adapters were pooled, purified by Mag-Bind kit, and size selected with a Pippin Prep 1.5 % agarose gel cassette (Sage Science, Beverly, MA, USA) for fragment sizes of 250–500 bp. PCR enrichment was performed in 18 cycles in ten 10 μL reactions per sublibrary with Phusion HF PCR Mastermix (New England BioLabs) by mixing 5 µL of 2× Phusion Master mix, 0.4 µm of each standard Illumina P1-i5 (5ʹ-AATGATACGGCGACCACCGA-3ʹ) and P2-i7 (5ʹ-CAAGCAGAAGACGGCATACGA-3ʹ) PCR primer, 3.2 µL of H_2_O and 1 µL of restricted-ligated DNA pool. Each sublibrary was purified with the Mag-Bind kit or 1.2× SPRIselect beads (Beckman Coulter, Brea, CA, USA) and quantified on Qubit. All sublibraries were pooled equimolarly and sequenced on the NovaSeq 6000 platform (Illumina) using the SP reagent kit v.1.5 in 2 × 150 bp configuration at the Institute of Experimental Botany, Czech Academy of Sciences, Olomouc, Czech Republic.

### Double-digest restriction site-associated DNA sequencing data analysis

Raw reads were checked for quality in FastQC v.0.11.9 (https://www.bioinformatics.babraham.ac.uk/projects/fastqc) and demultiplexed in Stacks v.2.63 (Catchen *et al.*, 2013). SeqPurge v.2019_09 (Sturm *et al.*, 2016) was used for quality filtering and adapter trimming with default settings. Reads were subsequently mapped to the reference whole-genome sequence of *R. moschus* using the BWA-MEM algorithm (bio-bwa.sourceforge.net) and converted to BAM files via Samtools (Li *et al.*, 2009). A catalogue of loci was created in the Gstacks module of Stacks and subsequently used for data filtering in the Populations module. Alternative *de novo* and reference-based pipelines with tetraploid settings were also tested in ipyrad (Eaton and Overcast, 2020), but these resulted in fewer recovered loci and/or a more noisy phylogenetic signal (not shown) and were, therefore, not used further.

For *R.* ser. *Glandulosi* populations and ancestral taxa, diversity indices [heterozygosity, the inbreeding coefficient (*F*_IS_) and the number of private alleles] were calculated in Populations with the *R* parameter (minimum proportion of individuals to process a locus) set to 0.5. To assess the correlation between genetic diversity indices and the distance from the borderline between sexuals and apomicts (sex–apo borderline *sensu*  Sochor *et al.*, 2024; Fig. 2), the distance was measured for each sexual individual using the Distance-to-nearest hub function in QGIS v.3.30.0 (www.qgis.org), and the expected heterozygosity (*H*_exp_) was calculated for each individual sample in Populations with *R* = 0.7 and a minimum minor allele frequency required to process a nucleotide site (min-maf) = 0.05. Linear regression was subsequently calculated in NCSS v.9 (www.ncss.com). Given that *H*_exp_ was significantly correlated with data coverage at the individual level, linear regression was also calculated with residuals of *H*_exp_ from its regression on the number of the recovered variant sites. Populations Fra, Pyr, Ape, Cau and Hyr were excluded from these analyses owing to unclear placement of the borderline in Western Europe and geographical isolation of the Asian populations.

Private alleles were identified in Populations for each of the potentially ancestral species (diploids and *R. caesius*; Table 1) and subsequently traced using our custom-made script (available at github.com/hajnej/population_private_alleles) in the target (derived) individuals in populations.sumstats.tsv generated by another Populations run. Numbers of private alleles detected in each individual were divided by the total number of variant sites for the individual to avoid the effects of unequal coverage and missing data. Alleles identified as private for *R.* subsect. *Rubus* against the extant potentially ancestral species were considered as originating in the extinct *Suberecti* ancestor; these alleles were traced separately owing to the hybrid origin of the *R.* subsect. *Rubus* polyploids.

To obtain another proxy for allele sharing between ancestral and derived taxa, a reference sequence for each potentially ancestral taxon (Table 1) was constructed from double-digest restriction site-associated DNA sequencing (ddRADseq) reads (hereafter termed ‘pseudoreference’ to avoid confusion with the *R. moschus* whole-genome reference sequence); reads from the ancestral taxa were used for *de novo* assembly in ipyrad (clustering threshold 0.9, other parameters left as the default), consensus sequences of all samples within species were mixed together, and microbial contaminants were excluded by BBDuk (sourceforge.net/projects/bbmap) by mapping to the available viral, bacterial, fungal and protozoan reference genomes from the NCBI Reference Sequence Database. Reads of each individual of the derived taxa were mapped by BWA-ALN (bio-bwa.sourceforge.net) with *maxDiff* set to one, and the proportion of mapped reads was calculated in Gstacks as a proportion of all reads that were not unmapped (i.e. including those with low mapping qualities and soft-clipped alignments). To correct for inter-individual variation in data quality (and thus read mapping), the average mapping efficiency across pseudoreferences within an individual was subtracted from each value.

Structure v.2.3.4 (Pritchard *et al.*, 2000) was used for inferring population genetic structure and ancestry for two sample sets: (1) the *Basic set* was composed of all samples except *R. incanescens* (owing to small sample size), *R. idaeus*, *R. caesius* and its three hybrid derivatives (owing to their apparently negligible role in the evolution of *R.* ser. *Glandulosi* and to reduce complexity of the dataset) and polyploid outgroups (for their complex origin), but included *R.* ser. *Rubus* and North American taxa (representatives of *R.* subsect. *Rubus*); and (2) the *European set* further excluded all extra-European samples. The following set-up was used for the computation: admixture model, no linkage, no prior population information, number of clusters *K* in the range of three to seven in ten replicate runs for each *K*, with 150 000 burn-in iterations followed by a further 200 000 Markov chain Monte Carlo iterations. Stacks parameters for the input data filtering were set as follows: *R* = 0.7, min-maf = 0.05 and one random single nucleotide polymorphism (SNP) per locus. The best value of *K* was selected using the method of Evanno *et al.* (2005) as implemented in StructureHarvester (Earl and vonHoldt, 2012) and according to similarities among runs and interpretability of the results. Clumpak (Kopelman *et al.*, 2015) was used for post-processing and visualization of the results.

RADpainter and fineRADstructure v.0.3.3 (Malinski *et al.*, 2018) analyses were performed with another two sample sets: (3) the *Complete sample set* (including all polyploid and diploid outgroups); and (4) the *European Glandulosi set* (i.e. excluding all outgroups and Asian *Glandulosi* samples, in addition to 25 samples with >80 % of missing data). The analyses included calculation of the coancestry matrix, assignment of individuals into clusters (admixture model with burn-in of 50 000 or 250 000 for the two datasets, respectively, followed by 100 000 or 500 000 Markov chain Monte Carlo iterations with the thinning interval of 1000) and the maximum a posteriori (MAP) tree building (with the same number of iterations). The associated plotting R script fineRADstructurePlot.R was used for generating coancestry heatmaps. Populations parameters for the input data filtering were set at *R* = 0.5, min-maf = 0.05.

### Present-day and palaeoclimatic distribution modelling

Models of suitable climate were computed for the tetraploid *R.* ser. *Glandulosi* using present-day bioclimatic variables from WorldClim 1.4 (Hijmans *et al.*, 2005), with a spatial resolution of 2.5 arc-min. Owing to the large overlap of ecological niches of tetraploid sexuals and apomicts (Sochor *et al.*, 2024), the two reproductive groups were treated together as a single taxon. Given that the occurrence records were spatially biased towards Central Europe, occurrences closer to each other than a minimum distance of 30 km were removed, using spThin library (Aiello-Lammens *et al.*, 2015), and the resulting 192 occurrences were used in the modelling.

Because strong collinearity between bioclimatic variables might reduce model predictive ability (Svenning *et al.*, 2011), confuse model interpretation (Baldwin, 2009) and reduce model transferability (Warren *et al.*, 2014), we used a combination of selecting bioclimatic variables relevant to the ecological and physiological processes determining species distribution with a reduction of the number of variables through a statistical analysis. To assess multicollinearity between variables, 10 000 random points were generated within the minimal convex polygon around the thinned records plus a buffer of 2 arc-min to extract cell values for all the variables. The resulting matrix was analysed using the vifcorr function from the usdm library (Naimi *et al.*, 2014) to find strongly correlated pairs of variables. If the Pearson correlation coefficient was higher than |0.85| for a pair of variables, only one of them with a supposed tighter relationship with plant ecology was retained. At the end, six bioclimatic variables were selected: Bio2 (mean diurnal temperature range), Bio3 (isothermality), Bio5 (maximum temperature of warmest month), Bio6 (minimum temperature of coldest month), Bio12 (annual precipitation) and Bio15 (precipitation seasonality).

Occurrence records and environmental data were used to calibrate the distribution model using the MaxEnt method (Phillips and Dudík, 2008). This machine-learning method was selected because it generally performs well under different scenarios when only presence data are available (Elith *et al.*, 2006). To build a suite of candidate models with differing constraints on complexity and to quantify their performance, MaxEnt models were built and evaluated using the semi-automated streamline analysis in wallace v.2.1 (Kass *et al.*, 2023), which calls internally maxnet v.0.1.4 (cran.r-project.org/web/packages/maxnet/) and ENMeval v.2.0 (Kass *et al.*, 2021). Models used 10 000 background points generated within the buffer area of 2 arc-min around thinned records to extract cell values for the selected variables. We applied a spatial data partitioning scheme (Checkerboard 2 with *k* = 4 groups; Muscarella *et al.*, 2014), split occurrence data into training and validation subsets, tested all feature classes and their combinations, in addition to a range of regularization multipliers (from 0.5 to 4.0, in increments of 0.5), and clamping procedure was accounted for (Phillips *et al.*, 2009). To find the best model, we used primarily delta *AICc* (Warren and Seifert, 2011), but also estimated other evaluation metrics calculated by wallace v.2.1: *AUC* for both training and validation subsets, *AUCdiff* (differences between the training and validation *AUC*s), and the omission rate (*OR10pct*, i.e. 10 pct is the lowest suitability score for such localities after excluding the lowest 10 % of them).

The final model computed for present-day climate was run in the standalone MaxEnt application v.3.4.4 (Phillips and Dudík, 2008) and used a 10-fold cross-validation procedure and a clog-log output. The final model was projected to two palaeoperiods: Last Glacial Maximum (LGM; 22 000 years BP) and mid-Holocene (6000 years BP). The same bioclimatic variables and resolution as used for present-day climate were used for both palaeoperiods, and variables were taken from two palaeoclimatic models (CCSM4; Gent *et al.*, 2011; and MIROC-ESM; Watanabe *et al.*, 2011) of WorldClim 1.4 downscaled palaeoclimate (Hijmans *et al.*, 2005). Final maps were constructed in QGIS, using Google Satellite as a background map. Masks of LGM ice sheets and glaciers were extracted from Last Glacial Maximum v.1.0.1 compiled by Zentrum für Baltische und Skandinavische Archäologie (ZBSA; zbsa.eu/en/last-glacial-maximum), those of LGM continuous and discontinuous permafrost from Lehmkuhl *et al.* (2020), and LGM palaeocoastlines from Zickel *et al.* (2016).

## RESULTS

### *Rubus moschus* whole-genome reference sequencing

Illumina sequencing provided 408.8 million reads (61.7 Gbp of total length, a quality score of 20 (Q20) reached in 97.6 % of reads). After plastome and low-quality reads filtering, 1.54 million ONT reads (8.1 Gbp of total length, N50 = 9655 bp) were retained. The *de novo* assembly after polishing resulted in 2170 contigs (plus one plastome contig) with a total length of 314 423 542 bp and N50 of 326 641 bp. The scaffolded assembly contained seven chromosome contigs (290.6 Mbp in total) and 511 unplaced contigs (24.0 Mbp), with N50 of 39 737 832 bp. BUSCO analysis identified 97.6 % of complete (89.4 % single-copy, 8.2 % duplicated), 0.6 % of fragmented and 1.8 % of missing genes. The final sequence is available at the Mendeley Data repository (DOI: 10.17632/c7x3pf4b72.1).

### Genetic diversity and its spatial structuring

Among the *Glandulosi* populations, no marked differences were detected in both expected and observed heterozygosity (Table 2). The *F*_IS_ index was influenced only by sample sizes. For example, the apomictic population (Apo) exhibited markedly higher *F*_IS_, but after a random selection of 30 samples, it dropped to a value only slightly higher than those of the sexual populations of the same size (*F*_IS_ = 0.078), with only a negligible change in observed heterozygosity and *H*_exp_ (0.030 and 0.047, respectively). The absolute number of private alleles was also associated with sample size, but after correction by the number of samples per locus (*PA*/*N* per locus), the values varied only from 191 to 347 in European populations, while the Asian populations Cau and Hyr showed values of 809 and 795, respectively.

**Table 2.** Population genetic statistics based on 30 078 loci with 561 810 variant sites.

	*N*	*N* per locus	s.d.	*H*_obs_	s.d.	*H*_exp_	s.d.	*F*_IS_	s.d.	*PA*	*PA/N* per locus
Apo	159	104.0	21.0	0.028	0.067	0.048	0.103	0.154	0.284	23 679	227.7
Alp	31	22.5	4.3	0.030	0.079	0.043	0.101	0.067	0.218	6245	277.4
Ape	30	23.7	3.9	0.031	0.079	0.045	0.102	0.070	0.222	6625	279.7
Bal	32	25.6	4.1	0.030	0.079	0.041	0.099	0.063	0.212	8654	338.1
Fra	6	4.5	1.1	0.031	0.111	0.036	0.108	0.023	0.159	659	145.8
Her	36	23.4	4.7	0.028	0.077	0.041	0.100	0.066	0.215	4379	187.0
Pan	10	6.8	1.8	0.029	0.092	0.041	0.107	0.041	0.193	2200	325.2
Pyr	17	10.3	2.4	0.030	0.086	0.044	0.109	0.054	0.210	1970	190.9
SEC	32	20.2	4.3	0.027	0.074	0.041	0.099	0.070	0.227	7016	346.9
WCa	30	22.5	3.7	0.029	0.080	0.040	0.099	0.057	0.203	5532	246.0
Cau	31	21.0	3.9	0.028	0.078	0.039	0.097	0.058	0.209	17 021	808.7
Hyr	24	15.7	3.7	0.028	0.082	0.041	0.107	0.057	0.208	12 498	795.4
*R. caesius*	15	7.2	3.3	0.045	0.130	0.066	0.149	0.066	0.241	29 650	4140.9
*R. canescens*	15	10.4	3.3	0.016	0.064	0.027	0.092	0.038	0.178	7002	672.1
*R. dolichocarpus*	25	14.6	5.4	0.012	0.052	0.025	0.089	0.044	0.186	12 081	828.9
*R. idaeus*	5	3.0	1.3	0.016	0.091	0.023	0.095	0.024	0.155	21 451	7073.5
*R. incanescens*	4	3.7	0.7	0.019	0.108	0.017	0.081	0.003	0.107	5678	1534.7
*R. moschus*	8	5.3	1.7	0.012	0.064	0.020	0.083	0.022	0.141	2866	536.8
*R.* subsect. *Rubus*	14	9.8	3.2	0.039	0.102	0.063	0.132	0.079	0.255	36 822	3753.6
*R. ulmifolius* agg.	27	20.1	6.7	0.021	0.064	0.042	0.108	0.077	0.240	19 281	961.6

Abbreviations: *F*_IS_, inbreeding coefficient; *H*_exp_, expected heterozygosity; *H*_obs_, observed heterozygosity; *N*, number of individuals; *PA*, number of private alleles; s.d., standard deviation of the preceding statistics.

**Fig. 3.**
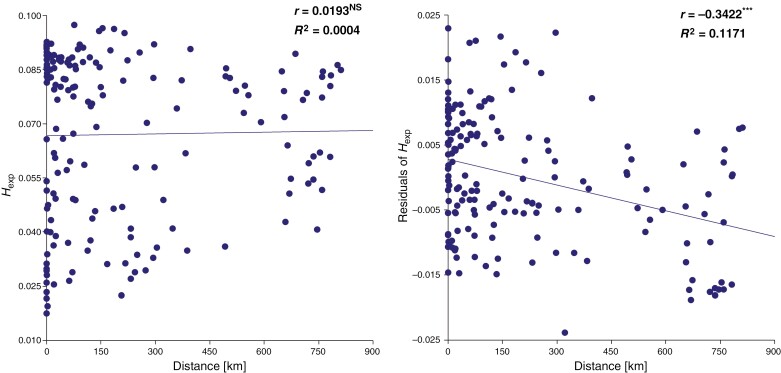
Linear regression of expected heterozygosity of individual genotypes (*H*_exp_; left) or its residuals (from linear regression of *H*_exp_ on the number of recovered variant sites; right) on the distance from the sex–apo borderline (Distance); only Central and Southeast European sexual individuals (excluding Fra, Pyr, Ape, Cau and Hyr) with >6000 variant sites were included (169 individuals); the analysis is based on 15 581 loci with 19 643 variant sites.

There was no correlation between the genetic diversity (*H*_exp_) of sexual individuals and their distance from the sex–apo borderline when analysed directly. However, a significant negative correlation was found when using residuals of *H*_exp_ from its regression on the number of variant sites (Fig. 3).

### Private allele tracing and mapping of reads to pseudoreferences

**Fig. 4.**
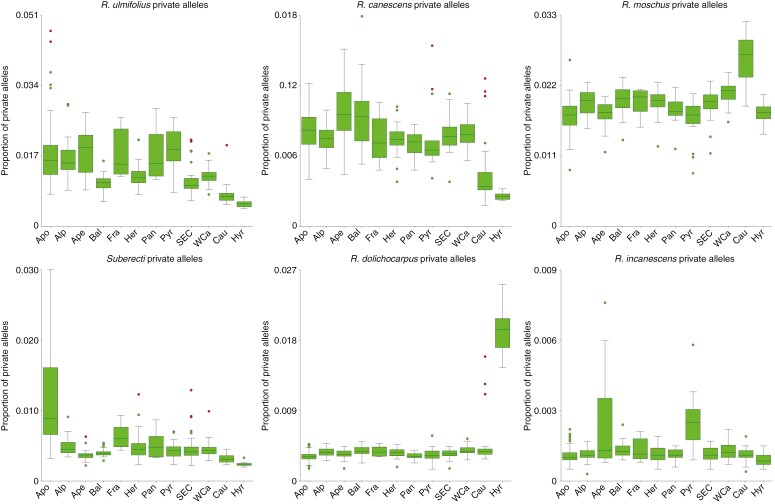
Proportion of private alleles of ancestral taxa detected in the *Glandulosi* populations: apomicts (Apo), Alps (Alp), Apennines (Ape), Balkans (Bal), France (Fra), Hercynia (Her), Pannonia (Pan), Pyrenees (Pyr), Southeastern Carpathians (SEC), Western Carpathians (WCa), Caucasus (Cau) and Hyrcania (Hyr). For the full dataset (including non-*Glandulosi* taxa, and *R. caesius* and *R. idaeus* private alleles), see Supplementary Data Fig. S2. Boxes indicate 25th, 50th and 75th percentiles; whiskers show the largest/smallest observation that is less than or equal to the upper/lower edge of the box plus 1.5 times the box height; mild outliers (multiplier 1.5) are shown as green dots and severe outliers (multiplier 3.0) as red dots.

Although the read mapping generally provided a somewhat weaker signal, the two approaches provided congruent results and independently revealed the following dominant patterns of introgression (Fig. 4; Supplementary Data Figs S1 and S2). Markedly elevated values of private alleles and read mapping efficiencies were detected in the Hyrcanian population (Hyr) in relation to *R. dolichocarpus*. This was also the case for the three Caucasian samples (Cau) from Kakheti (easternmost Georgia with the common occurrence of this diploid), but not for the other Caucasian individuals from western regions. Those, in contrast, exhibited a significantly increased affinity to *R. moschus*. The affinity to *R. ulmifolius* agg. was decreased in the Asian populations, but to a lesser extent also in the Balkan, Carpathian and Hercynian populations (Bal, SEC, WCa and Her), whereas all the other populations exhibited higher values but also with higher variability. Increased affinity to *R. canescens* was detected in the populations Bal and Ape (particularly its southern subpopulation), but also with rather high variability. Significantly increased affinity to *R.* subsect. *Rubus* (*Suberecti*) was detected in apomicts (Apo) and to a lesser extent in French sexuals (Fra) and three Pannonian (Pan) sexual individuals, in the latter always in association with increased affinity to *R. ulmifolius* agg. Searching for genetic traces of *R. incanescens* was slightly hindered by the availability of only four individuals from two localities of this species for analysis. However, increased proportions of its private alleles were detected in almost all north-Apennine (Ape) individuals and in most of the Pyrenean (Pyr) individuals. Increased affinity to *R. caesius* was detected in only three apomictic (Apo) individuals. Likewise, the affinity to *R. idaeus* was generally very low and with only a few outliers of unclear interpretation.

### Population structure and coancestry matrices

**Fig. 5.**
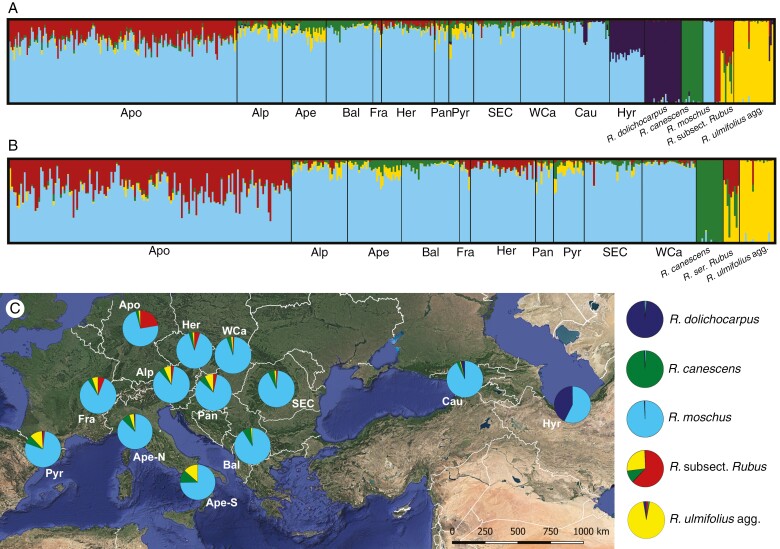
Population inference from Structure based on the *Basic sample set* (A, C; 7329 unlinked SNPs; *K *= 5) and the *European set* (B; 7596 unlinked SNPs; *K* = 4) visualized as bar charts for each individual (A, B) and pie charts with individual assignment probabilities averaged for each population or taxon (C). The pie charts in C are shown on the map of Europe and West Asia for each population (note that the Ape population has been split here into northern and southern subpopulations to show population subdivision), with ancestral taxa set aside. Each of the plots represents an average of ten runs (similarity score = 1.0). *Rubus* subsect. *Rubus* covers North American and European taxa (*R.* ser. *Rubus*) in this order. For the other *K* values, see Supplementary Data Figs S3 and S4. Background map layer: Google Earth.

Structure analysis resulted in the best-supported *K* = 4 in both sample sets (*Basic set* and *European set*), but *K* = 5 (the second best) provided a biologically more reasonable interpretation in the *Basic set*. All variants of the analysis distinguished clusters corresponding to the diploid taxa and the *Glandulosi* and *Suberecti* ancestors, the last of which was detected mainly in *Glandulosi* apomicts, in much lesser degree in sexuals (mainly population Her; Fig. 5). Small affinities to *R. ulmifolius* agg. were detected in populations Alp, Ape (mainly its southern part) and Pyr. Low levels of coancestry signal from *R. canescens* were revealed mainly in Ape and Bal, to a lesser extent also in Pyr and SEC. In contrast, the signal from *R. ulmifolius* agg. and *R. canescens* was mostly negligible in apomicts (except for about seven individuals). A clear coancestry was detected between *R. dolichocarpus* and the Hyr population (plus three Cau individuals from eastern Georgia). *Rubus moschus* clustered with polyploids of *R.* ser. *Glandulosi*; only for *K* = 7 it formed a separate cluster shared, in part, with the Cau and Hyr populations (Supplementary Data Fig. S3).

The fineRADstructure analyses indicated elevated coancestry mainly between Hyrcanian (Hyr; plus three Caucasian, Cau) sexuals and *R. dolichocarpus* and between Cau sexuals and *R. moschus*. Three apomictic *Glandulosi* clearly showed affinity to *R. caesius*; slightly elevated coancestry was also detected between Pyr and Ape and *R. incanescens*, and between part of *Glandulosi* apomicts and *R.* subsect. *Rubus* (and other non-*Glandulosi* apomicts; Supplementary Data Fig. S5). Depending on parameters for data filtering, sample selection and fineRADstructure settings, the tree-based population assignment (hence the grouping of samples in heatmaps) was rather unstable, probably owing to the reticulate nature of the studied system, but the resulting coancestry signal remained very similar across different analysis set-ups. Populations Cau and Hyr formed two separated groups from the European *Glandulosi* (yet with higher affinity to that group than to polyploid outgroups). They were, therefore, removed together with outgroups to reduce complexity.

**Fig. 6.**
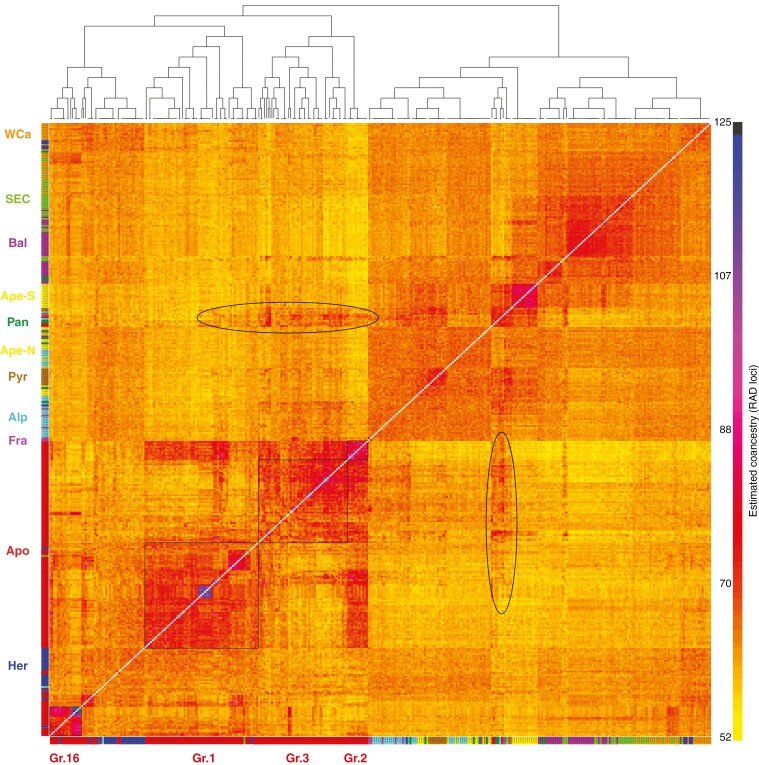
FineRADstructure coancestry matrix and maximum a posteriori (MAP) tree based on the *European Glandulosi set* and 43 444 loci with 102 621 SNPs. Colours representing populations are as in Fig. 2. The four groups of apomicts are marked as thin black squares. Increased coancestry, probably resulting from introgression from non-*Glandulosi* apomicts, is marked as ovals.

In the resulting *European Glandulosi set*, most of the sexuals formed a separate group (Fig. 6), except for most of the Her (and in some replications also WCa; not shown) individuals, which exhibited increased affinity both to other *Glandulosi* sexuals and apomicts, and their position was unstable. Within *Glandulosi* sexuals, a small, geographically diverse group of individuals exhibited a lower affinity to the other *Glandulosi* sexuals; this group also included three samples of *Glandulosi* apomicts and showed an increased affinity to *R. ulmifolius* agg. and non-*Glandulosi* apomicts (Supplementary Data Fig. S5), which most probably resulted from introgression from non-*Glandulosi* apomicts (marked in Fig. 6). The other sexuals were rather homogeneous and were constantly divided into two weakly differentiated clusters: the Balkan and Carpathian populations (Bal, WCa and SEC) and the western populations (Alp, northern Ape, Pyr and Fra). The southern part of the Ape population clustered constantly with the introgressed individuals, probably owing to their coancestry with *R. ulmifolius* agg., and exhibited elevated affinity to Bal and SEC, in addition to the western populations (Alp, Pyr and Fra).

*Glandulosi* apomicts tended to form four dominant, yet not always clearly delimited clusters. The most distinct one was Genotype 16 and its derivatives (Group 16), whose affinity to other apomicts was rather low. Group 1 apomicts shared their coancestry mainly with the Her sexuals and many individuals also with the WCa (and SEC and Bal) sexuals. Group 3 apomicts exhibited slightly elevated coancestry with the western sexuals Alp, Fra and Pyr. Apomicts of Group 2 exhibited markedly elevated coancestry with the Group 1 and 3 apomicts, but their affinity to *Glandulosi* sexuals (except for Her) was lower. Several individuals within Group 1 exhibited an elevated affinity to Group 3, and two individuals from Group 3 exhibited marked coancestry with Genotype 16 in all analyses. Virtually the same results were obtained even after removal of loci that contained private alleles of the ancestral species from analyses (not shown).

### Present-day and palaeodistribution modelling

The MaxEnt model of the present-day predicted distribution of tetraploids of *R.* ser. *Glandulosi* with linear, quadratic, hinge and product features (LQHP) and regularization parameter 1.0 was selected as the best in the model evaluation, based on the lowest *AICc*. Statistical validation suggested good model performance (*AUCtrain* = 0.838, *AUCvalid* = 0.820 ± 0.016, *AUCdiff* = 0.021 ± 0.016 and *OR10p* = 0.127 ± 0.035, mean ± s.d.). The mean *AUC* for the ten replicated runs was 0.779 ± 0.057. The highest mean relative contributions to the replicated models based on percentage contribution and permutation importance, respectively, were provided by Bio5 (maximum temperature of warmest month; 33.1 %/25.9 %), Bio6 (minimum temperature of coldest month; 26.8 %/18.7 %), Bio12 (annual precipitation; 21.8 %/20.7 %) and Bio15 (precipitation seasonality; 12.3 %/26.3 %) variables. In contrast, Bio2 (mean diurnal range) and Bio3 (isothermality) variables contributed little (always <6 %) to the models. Response and marginal curves of each bioclimatic variable are shown in Supplementary Data Fig. S6.

**Fig. 7.**
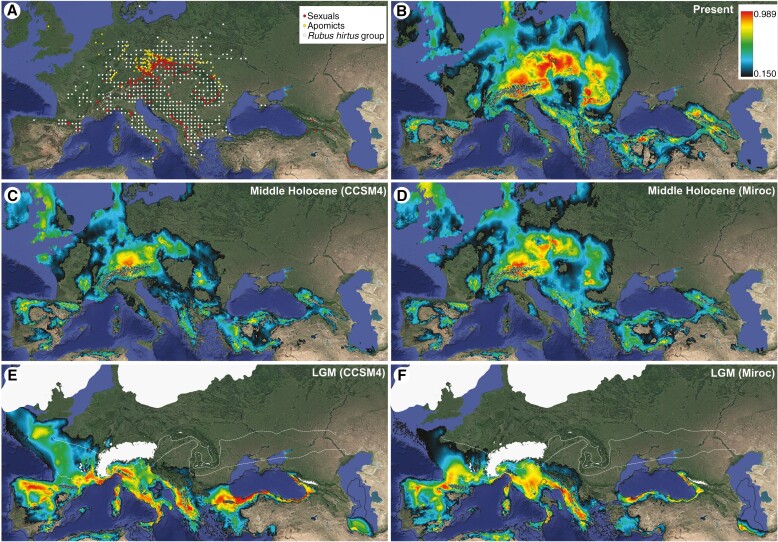
Distribution maps and climatic models. (A) Known distribution of *Rubus hirtus* group (Kurto *et al.*, 2010; white points) and localities of tetraploid apomicts (yellow points) and sexuals (red points) of *Rubus* ser. *Glandulosi* as published by Sochor *et al.* (2024). (B–F) Mean MaxEnt models of predicted distributions (clog-log output, mean of ten models each) for *R.* ser. *Glandulosi* (both apomictic and sexual) for the present, the mid-Holocene (6000 years BP) and the Last Glacial Maximum (LGM; 22 000 years BP). For past climates, projections on two climatic models are presented (CCSM4 in C, E; and Miroc in D, F), based on a distribution model computed with present-day climatic data. For the LGM, ice sheets and glaciers (white polygons), continuous and discontinuous permafrost (white dashed lines) and palaeocoastlines (black lines) are shown.

The mean MaxEnt model of the predicted distribution under the current climate fitted well with the real distribution of *R.* ser. *Glandulosi*. Only westernmost Europe was slightly underestimated, probably owing to the undersampling of this region, and the Baltic region was overpredicted in comparison to the real distribution of the tetraploids (Fig. 7A, B). The LGM models (Fig. 7E, F) predicted a suitable climate for the studied group almost exclusively in southern Europe (Iberian Peninsula, southern France, Apennine Peninsula, western and southern Balkans), western Asia Minor, along the southern and eastern Black Sea coast, and in Hyrcania. A comparison of two climatic scenarios of the LGM showed some regional differences in the predicted distribution mainly in Western Europe, western Asia Minor and the southeastern Balkans. The Mid-Holocene models (Fig. 7C, D) predicted a suitable climate mainly in Central Europe north of the Alps, but a somewhat more patchy distribution of suitable climate was predicted also elsewhere in Europe and West Asia. Only small patches were predicted in the Apennine Peninsula, Crimea and Hyrcania.

## DISCUSSION

### Sexuals are not genetically impoverished along the contact zone with apomicts

In our previous work (Sochor *et al.*, 2024), we concluded that the extraordinary borderline between tetraploid sexuals and apomicts of *R.* ser. *Glandulosi* probably resulted from phylogeographical history and neutral processes, because vast ecological niche overlap of sexuals and apomicts did not imply any role of (mal)adaptive (selection-dependent) factors in their geographical differentiation. However, the role of population genetic processes behind the observed spatial pattern, tentatively suggested already by Šarhanová *et al.* (2017), could not be ruled out. It is commonly presumed that marginal sexual populations tend to be small, fragmented, and impoverished in their genetic diversity (Sagarin and Gaines, 2002; Eckert *et al.*, 2008; Angert *et al.*, 2020). This can lead to decreased adaptability, susceptibility to genetic drift, inbreeding or outbreeding depression (the latter usually owing to secondary contact with related taxa), mutational meltdown, etc. (Keller and Waller, 2002; Willi *et al.*, 2013). Additionally, continuous gene flow from central to marginal populations can prevent local adaptations (García-Ramos and Kirkpatrick, 1997). Owing to their asexual reproduction, polyploidy and hybridity, apomicts can overcome or buffer such processes and gain an evolutionary advantage over the sexuals. Our ddRADseq did not reveal any differences among apomicts, sexual populations adjacent to the apomicts (mainly Her, WCa and Alp), and those further from their main range (Bal, Ape, Pan, SEC and Pyr) in both heterozygosity and inbreeding coefficient (Table 2). Furthermore, contrary to the expectation, the genetic diversity of individual genotypes (expressed as *H*_exp_) was negatively correlated with the distance from the range of apomicts (Fig. 3). This probably implied a gene flow from *Glandulosi* apomicts to sexuals rather than genetic impoverishment of the latter and reflected the ecological optimum of sexuals (Fig. 7) and large sizes and high density of their populations in Central Europe (the authors’ personal observation). Therefore, our data do not support the view that population genetic processes typical for genetically impoverished populations can drive the geographical parthenogenesis in *R.* ser. *Glandulosi*.

### *Rubus* ser. *Glandulosi* is introgressed by several taxa

*Rubus* ser. *Glandulosi* has always been regarded as one of the taxonomically most problematic *Rubus* taxa (Kurtto *et al.*, 2010). This fact stems not only from varying levels of sexuality, recently characterized as geographical parthenogenesis (Sochor *et al.*, 2024), but also from an extreme morphological variation and plasticity (‘amorphous’ taxon; Holub, 1997). Although a relatively low diversity of plastid haplotypes and ITS ribotypes was detected in the group in Europe (Sochor *et al.*, 2015), a distinct haplotype was detected in West Asia (Sochor and Trávníček, 2016; Kasalkheh *et al.*, 2024), which might imply a genetic contribution of an unknown (and probably extinct) ancestor in that region. Besides that, we detected at least some traces of each of the known Eurasian *Rubus* ancestors (see Table 1) in our *Glandulosi* ddRADseq data, except for *R. idaeus*. The most prominent shared ancestry was detected between the *Glandulosi* apomicts and *R.* subsect. *Rubus* (*Suberecti* ancestor; see below), and between *R. dolichocarpus* and *Glandulosi* sexuals across the range of *R. dolichocarpus*. *Rubus moschus* is likely to have contributed to the tetraploid gene pool twice: first, when it (or its predecessor) established the common tetraploid *Glandulosi* gene pool (light blue in Fig. 5; see also Sochor *et al.*, 2015); and second, in the later evolution in the Western Caucasus (Fig. 4; Supplementary Data Figs S1 and S3). *Rubus incanescens* has hitherto not been suspected to be an ancestor for polyploid brambles, probably owing to its restricted distribution and rather sparse occurrence (Sochor *et al.*, 2015). However, we have traced its alleles in *R.* ser. *Glandulosi* in the northern Apennines and the Pyrenees (Fig. 4), although in low quantities that did not enable a clear recognition of this ancestor in other analyses. It is, therefore, likely that *R. incanescens* has played some role in the evolution of the northwestern Mediterranean *Rubus* floras, although its extent cannot be estimated at present. The two remaining Eurasian diploids, *R. canescens* and *R. ulmifolius* agg., exhibited regionally elevated genetic signatures in *R.* ser. *Glandulosi* (Figs 4 and 5). The last known *Rubus* ancestor, the tetraploid *R. caesius*, was traced in only three apomictic individuals and appears to be of low importance in the *Glandulosi* evolution, because we were otherwise unable to confirm its ancient (cf. Sochor *et al.*, 2015) or contemporary involvement.

Although we detected a non-negligible gene flow from six diploid species (including the presumably extinct *Suberecti*), its mechanism across ploidy levels remains unclear. The mechanism of triploid bridge (Ramsey and Schemske, 1998) appears to be rather insignificant, because we have detected only one triploid hybrid of *R.* ser. *Glandulosi* and a diploid (*R. dolichocarpus*), and the hybrid was sterile, which was expected in a triploid hybrid of two sexuals. In contrast, two putative hybrids with *R. ulmifolius* from Italy (with no other species detected at the localities) proved to be tetraploid (not shown) and indicated the possibility of hybridization via a reduced gamete of *Glandulosi* and an unreduced gamete of the diploid. Alternatively, the detected gene flow can proceed via non-*Glandulosi* polyploids, which frequently co-occur with *R.* ser. *Glandulosi*, produce viable hybrids (Šarhanová *et al.*, 2012, 2017) and are themselves derived from the diploid ancestors (Sochor *et al.*, 2015). This mechanism is presumed to be dominant, e.g. for the gene flow from *R. ulmifolius* via derived polyploids to Pannonian *Glandulosi* (Pan), for two reasons: (1) *R. ulmifolius* agg. does not occur in that region (Kurtto *et al.*, 2010); and (2) the elevated frequency of *R. ulmifolius* agg. alleles in these individuals is associated with alleles of other ancestors, such as *R. canescens* and *Suberecti*. However, distinguishing between the two homoploid mechanisms is difficult in most other cases.

### What is the origin of apomictic *Glandulosi?*

Our ddRADseq analysis showed that all *Glandulosi* apomictic genotypes share a part of their genomes (here called *Suberecti* subgenome) with each other but not with sexuals (or to only a minor extent). At the same time, this gene pool is shared with non-*Glandulosi* apomicts, particularly with *R.* subsect. *Rubus*, including the North American taxa and *R.* ser. *Nessenses*. Both North American taxa and *R.* ser. *Nessenses* are supposed to have undergone prolonged isolation from other European brambles and are thought to share a common ancestor, here referred to as *Suberecti*, which is now extinct in Europe (see also Sochor *et al.*, 2015). If this shared ancestry were the product of the current hybridization of *Glandulosi* sexuals and non-*Glandulosi* apomicts, increased proportions of alleles of other ancestors would also be expected in the *Glandulosi* apomicts. Apomicts from most taxonomic *Rubus* series, including ser. *Hystrix*, *Anisacanthi*, *Vestiti* and *Pallidi*, exhibit an increased genetic affinity to *R. ulmifolius* agg., which is one of the main ancestral species (Supplementary Data Figs S1 and S2). As mentioned above, the increased proportion of both *R. ulmifolius* agg. and *Suberecti* alleles was also detected in several sexual or transitional individuals (*sensu*  Sochor *et al.*, 2024), which might represent occasional hybrids or introgressants of the Holocene origin (marked in Fig. 6). On the contrary, most *Glandulosi* apomicts are remarkably similar to the sexuals in their low affinity to *R. ulmifolius* agg. (Figs 4 and 5). Therefore, our data imply that the *Suberecti* subgenome was not obtained by *Glandulosi* apomicts from the modern *Rubus* taxa, but probably from the now extinct *Suberecti* ancestor in the preglacial period. Subsequently, the *Glandulosi* apomicts formed a distinct phylogeographical lineage independent of the sexual lineage, and the detected gene flow between the two lineages results only from their secondary contact. This scenario is also supported by the relative genotypic richness of apomicts, which is positively correlated with the distance from the contact zone with sexuals (Sochor *et al.*, 2024).

However, our personal observations suggest that the absolute number of apomictic genotypes is likely to be higher along the contact with sexual populations as a result of higher population densities. Consequently, it is expected that the sexual populations will play a role in generating novel apomictic genotypes. Increased genetic affinity between apomicts and the adjacent sexual populations (Alp, Her and WCa) confirms this expectation (Fig. 6). However, coherence of *Glandulosi* apomicts in phylogenomic clustering implies that the gene flow from sexuals to apomicts is probably not the primary driving force in the evolution of apomicts. Interestingly, the Structure results also indicate the reverse gene flow to a lesser extent, i.e. from *Glandulosi* apomicts to sexuals, particularly in the Her population (Fig. 5).

Four groups can be distinguished tentatively in *Glandulosi* apomicts (Fig. 6). Group 3 has a very large distribution range (Supplementary Data Fig. S7) and also includes a few pentaploids (including *R. nigricans*). Group 2 has a narrow Central European range with a single genotype in the west (determined to be *R. serpens*/*angloserpens*). Group 1 has a Central to Eastern European distribution. The fourth group is formed around Genotype 16, which is possibly the most widespread tetraploid genotype in *R.* ser. *Glandulosi* (Sochor *et al.*, 2024). This genotype apparently served as an ancestor for many derived genotypes of smaller distribution, the other ancestors being Her/WCa sexuals or (in two detected cases) the Group 3 apomicts. It is unclear whether this grouping reflects their distinct origin (e.g. from isolated glacial refugia) or whether it is only artificial and mirrors different patterns of introgression. Considering the weak differentiation (Fig. 6; not detected in Fig. 5), the latter appears more likely. Group 2 shows strong coancestry to both Groups 1 and 3 but decreased coancestry to sexuals (except for Her) and might represent either ‘genetically pure’ *Glandulosi* apomicts or hybrids of Groups 1 and 3. Group 1 is clearly related to Central European sexuals (Her and partly also WCa), whereas Group 3 has the highest affinity to western populations (Alp and Pyr).

### Apomictic *R.* ser. *Glandulosi* might have migrated from two glacial refugia

We modelled a suitable climate for tetraploid *R.* ser. *Glandulosi* at two extreme time points in the recent geological past: the LGM and the mid-Holocene. During the first (cold and dry) extreme, *R.* ser. *Glandulosi* was probably restricted to southern refugia, in a similar manner to other nemoral species, because the models correspond well to the modelled distributions of *Fagus sylvatica* and *Carpinus betulus* (Svenning *et al.*, 2008), for example. Although the two climatic models used differ in predictions in several regions, *R.* ser. *Glandulosi* was probably able to survive the LGM in large continuous areas across southern Europe and in a more fragmented manner in West Asia (Fig. 7). The current distribution of *R.* ser. *Glandulosi* sexuals suggests their main LGM refugia in the Balkans and the Apennines/southern Alps. This is consistent with relatively low genetic differentiation (Figs 5 and 6) and low numbers of private alleles in European populations compared with those in the Caucasus and Hyrcania (Table 2), which were (and still are) more isolated (Fig. 7). Highly suitable climate was also predicted in southern France, along the southern margin of discontinuous permafrost (Fig. 7E, F). This region is the best candidate for the main glacial refugium of *R.* ser. *Glandulosi* apomicts owing to the slightly increased coancestry of the apomictic Group 3 and the western sexuals (Alp and Pyr). These apomicts are widely distributed from France to Romania (Supplementary Data Fig. S7). Therefore, this group of likely western origin might be ancestral to Central and Eastern European apomicts. Its survival north of the Alps is unlikely (Müller *et al.*, 2003; Binney *et al.*, 2017; Janská *et al.*, 2017) and unsupported by our climatic models. However, southern France might have provided suitable conditions during the LGM, because mesophilous tree species were recorded in the lower Rhone basin or the Pyrenean valleys (Delhom and Thiébault, 2005; Beaudouin *et al.*, 2007) or even at somewhat higher latitudes in the Landes de Gascogne (Svenning *et al.*, 2008; de Lafontaine *et al.*, 2014). From these regions, apomicts might have spread along the Alps to Central Europe, which appears to have been the most suitable region climatically in warm and moist periods of the Holocene. In contrast, areas with a suitable climate became more fragmented and restricted to high mountains in southern Europe (Fig. 7C, D), similar to the current state.

This scenario of spread of apomicts, however, does not appear to be likely for Genotype 16, which shows a low coancestry with the other apomicts and western sexuals, but exhibits a higher affinity to eastern sexuals, particularly several WCa and SEC individuals. Its extensive distribution area, covering the whole of Central Europe, from Czechia to Romania, and its apparent role in the formation of other apomictic genotypes suggest that it is an old genotype, probably of different phylogeographical history. Our models do not support its large-scale survival during the LGM in the Carpathians. However, the southern foreland of the Western Carpathians demonstrably hosted a woodland biota in protected and relatively moist valleys (Ložek, 2006; Jankovská and Pokorný, 2008). The same situation probably existed in the Eastern Carpathians, with a possible regional survival of deciduous trees (Magyari *et al.*, 2014; see also Molnár *et al.*, 2023). The survival of one generalistic apomictic genotype is therefore plausible. Such a scenario would imply that *R.* ser. *Glandulosi* apomicts were widespread in Central Europe also in the previous interglacial period and later contracted into two (or more) glacial refugia. Because the area of possible survival of Genotype 16 is now inhabited by sexuals (Fig. 2; Sochor *et al.*, 2024), this scenario would also necessarily imply that the contact zone is not stable and that apomicts are systematically edged out by sexuals, e.g. by the mechanism of clonal turnover (Janko, 2014). Alternatively, Genotype 16 might have been formed *de novo* in the Holocene from Carpathian sexuals completely independent of the other *Glandulosi* apomicts. This is, however, unlikely without a contribution of non-*Glandulosi* apomicts, whose genetic footprints were not detected in our ddRADseq data (Supplementary Data Fig. S5), nor are they suspected from morphology. At the same time, the *Suberecti* subgenome does not deviate between Genotype 16 and other *Glandulosi* apomicts (probabilities of assignment of Genotype 16 to the *Glandulosi*:*Suberecti* cluster being constantly around 86:14, with no other cluster assigned; Fig. 5).

## Conclusions

*Rubus* ser. *Glandulosi* is a widely distributed taxon with a complex evolutionary history, which resulted in a specific pattern of geographical parthenogenesis. The whole group is introgressed from six species (or seven, considering the unknown Caucasian ancestor inferred from plastid haplotypes; see the second subsection of the Discussion), but the gene pool of a single, extinct species, *Suberecti*, is characteristic for the apomicts and appears to be determinative for their reproductive mode. A fast postglacial recolonization of Central and Western Europe by *Glandulosi* apomicts from the main western refugium and, possibly, another small eastern refugium, accompanied by migration of sexuals from the Balkans and the Apennines/southern Alps probably established the contact zone between the reproductive modes. How stable this line of contact might be and which factors play a role in its shifts is unclear. Given that the available data do not suggest any differences in competitive abilities between sexuals and apomicts (M. Konečná, M. Sochor, M. Duchoslav, unpublished observations), we hypothesize that sexuals should gradually extend their distribution into the range of apomicts owing to the neutral process of clonal turnover, while the speed of this shift is determined by genotypic diversity and the population density of apomicts (Sochor *et al.*, 2024). In such a case, fast recolonization of Central Europe from the west might have been crucial for establishing a relatively stable position of the apomicts that guarantees resistance against the expansion of sexuals.

## SUPPLEMENTARY DATA

Supplementary data are available at *Annals of Botany* online and consist of the following.

Table S1: collection data of the analysed specimens, codes used in FineRADstructure analyses, and assignment into apomictic groups. Figure S1: boxplots of relative mapping efficiencies of reads to pseudoreferences of ancestral taxa. Figure S2: proportion of private alleles of ancestral taxa detected in *Rubus* ser. *Glandulosi* populations. Figure S3: population inference from Structure based on the *Basic sample set* and 7329 unlinked SNPs; *K* = 3–7. Figure S4: population inference from Structure based on the *European dataset* and 7596 unlinked SNPs; *K* = 3–6. Figure S5: FineRADstructure coancestry matrix visualized as a heatmap based on a complete sample set with 30 328 loci (98 362 variant sites). Figure S6: (A) Response curves showing how each environmental variable affects the MaxEnt prediction. (B) Marginal response curves; each of the curves represents a different model, namely, a MaxEnt model created using only the corresponding variable (mean ± s.d. from ten runs). Figure S7: geographical distribution of apomictic groups as defined by fineRADstructure analysis.

mcae050_suppl_Supplementary_Figures_S1-S7

mcae050_suppl_Supplementary_Table_S1
