# Supplementary material for: Plant kleptomaniacs: geographical genetic patterns in the amphi-apomictic Rubus ser. Glandulosi (Rosaceae) reveal complex reticulate evolution of Eurasian brambles
Source: Ann Bot. 2024 Mar 29;134(1):163–78. doi: 10.1093/aob/mcae050 (PMC11161565; doi:10.1093/aob/mcae050)
Supplement: mcae050_suppl_Supplementary_Figures_S1-S7 [file mcae050_suppl_supplementary_figures_s1-s7.docx]

Sochor et al.: Plant kleptomaniacs: geographic genetic patterns in the amphi-apomictic *Rubus* ser. *Glandulosi* (Rosaceae) reveal complex reticulate evolution of Eurasian brambles

**Supplementary data**

**Figure S1**


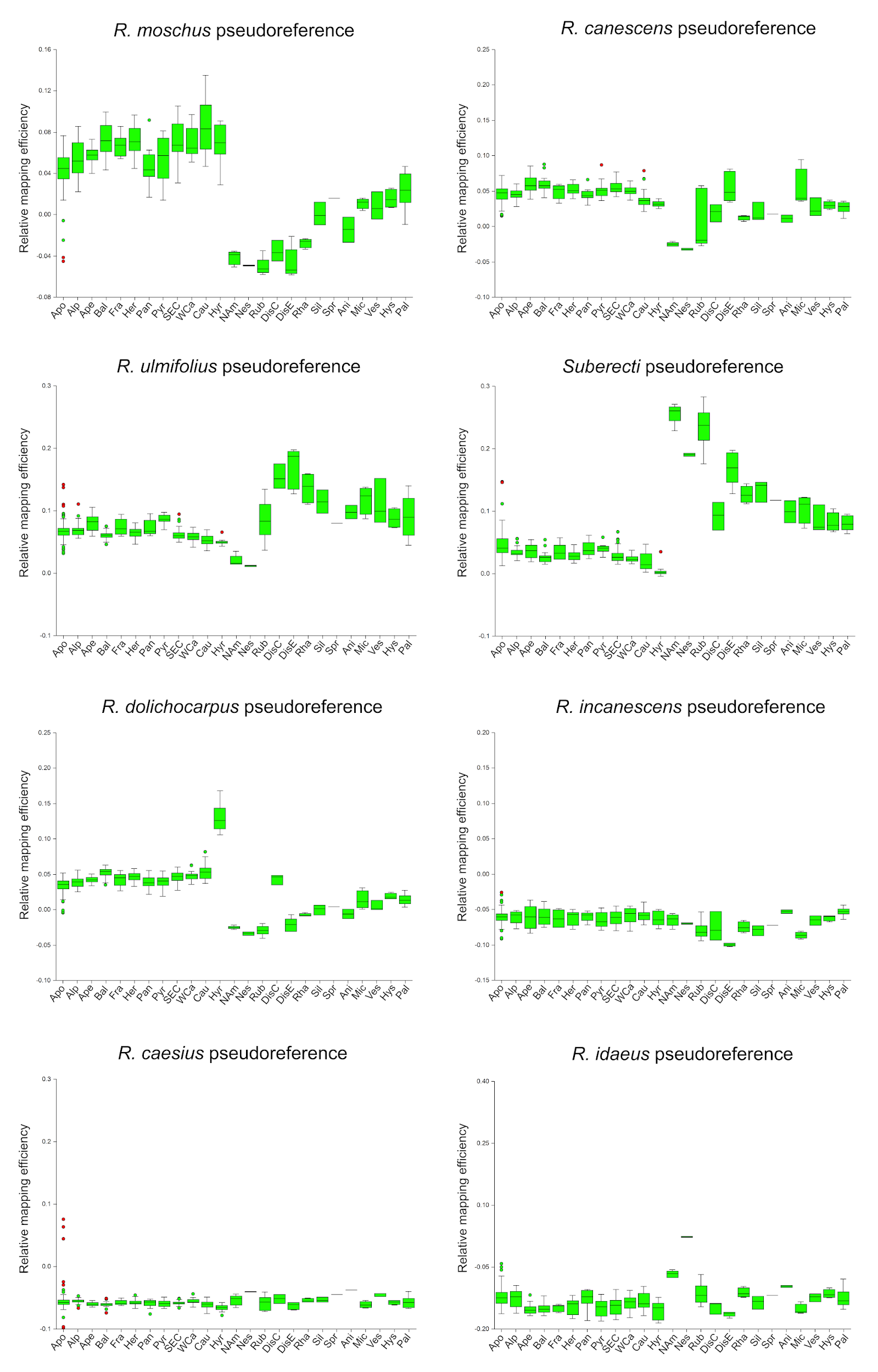


**Fig. S1:** Boxplots of relative mapping efficiencies of reads to psedoreferences of ancestral taxa. *Rubus* ser. *Glandulosi* populations: apomicts (Apo), Alps (Alp), Apennines (Ape), Balkans (Bal), France (Fra), Hercinia (Her), Pannonia (Pan), Pyrenees (Pyr), South-eastern Carpathians (SEC), Western Carpathians (WCa), Caucasus (Cau), Hyrcania (Hyr); outgroups: North American taxa (NAm), *R.* ser. *Nessenses* (Nes), *R.* ser. *Rubus* (Rub), *R.* ser. *Discolores* from the Caucasus and Europe (DisC, DisE, respectively), *R.* ser. *Rhamnifolii* (Rha), *R*. ser. *Silvatici* (Sil), *R.* ser. *Sprengeliani* (Spr), *R*. ser. *Anisacanthi* (Ani), *R*. ser. *Micantes* (Mic), *R.* ser. *Vestiti* (Ves), *R.* ser. *Hystrix* (Hys), *R.* ser. *Pallidi* (Pal).

**Figure S2**


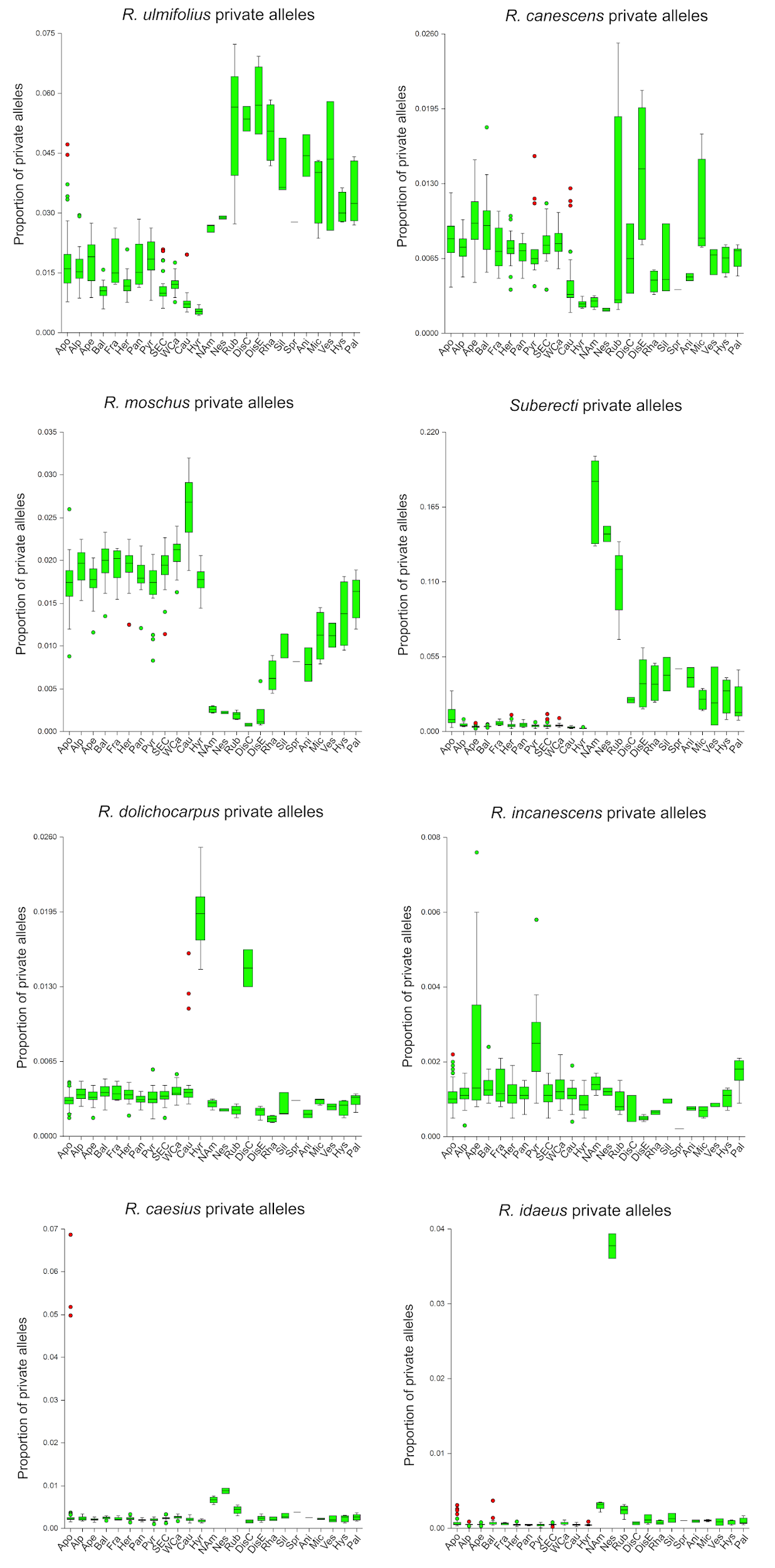


**Fig. S2:** Proportion of private alleles of ancestral taxa detected in *Rubus* ser. *Glandulosi* populations – apomicts (Apo), Alps (Alp), Apennines (Ape), Balkans (Bal), France (Fra), Hercinia (Her), Pannonia (Pan), Pyrenees (Pyr), South-eastern Carpathians (SEC), Western Carpathians (WCa), Caucasus (Cau), Hyrcania (Hyr); and outgroups – North American taxa (NAm), *R.* ser. *Nessenses* (Nes), *R.* ser. *Rubus* (Rub), *R.* ser. *Discolores* from the Caucasus and Europe (DisC, DisE, respectively), *R.* ser. *Rhamnifolii* (Rha), *R*. ser. *Silvatici* (Sil), *R.* ser. *Sprengeliani* (Spr), *R*. ser. *Anisacanthi* (Ani), *R*. ser. *Micantes* (Mic), *R.* ser. *Vestiti* (Ves), *R.* ser. *Hystrix* (Hys), *R.* ser. *Pallidi* (Pal).

**Figure S3**


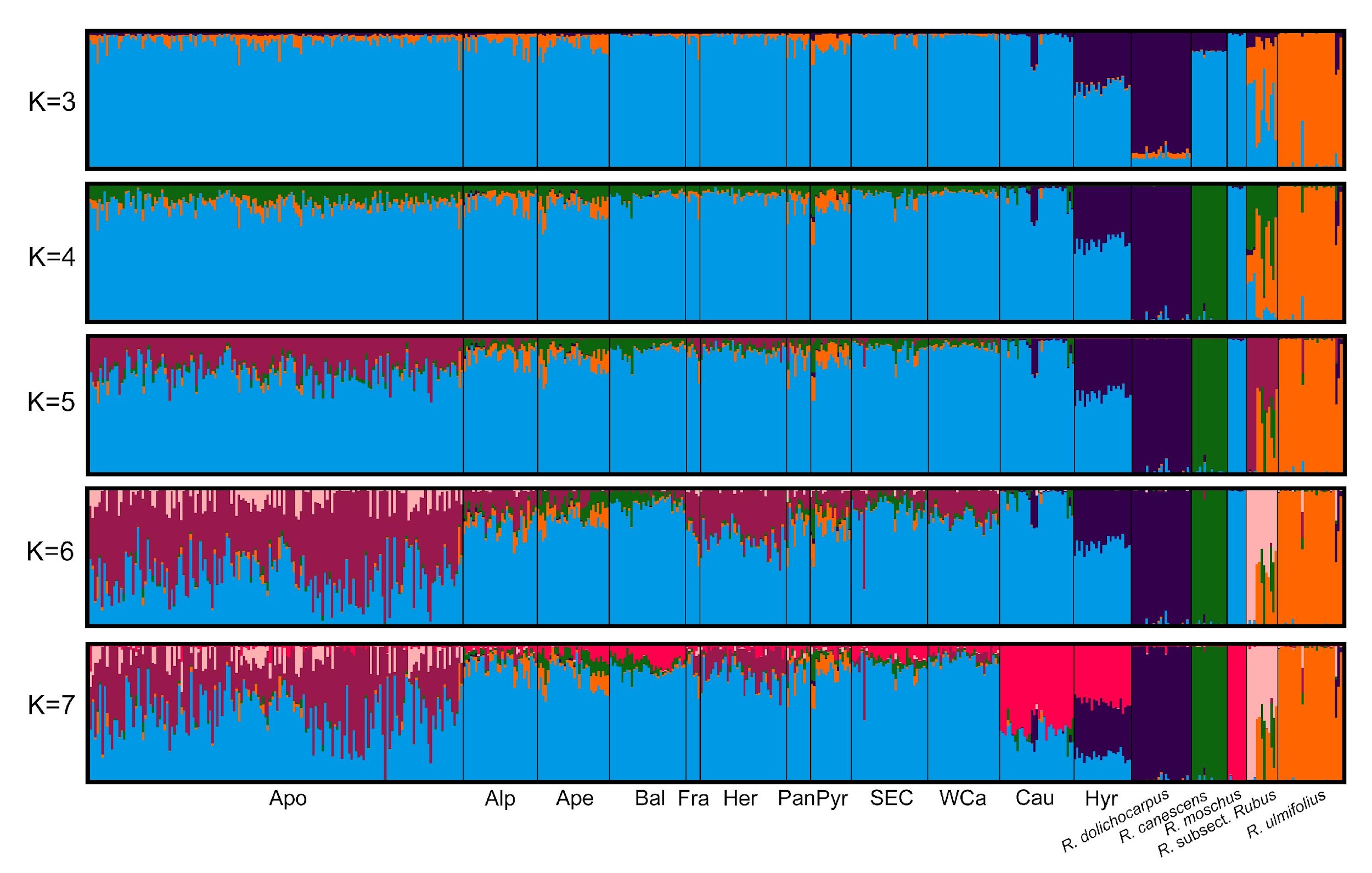


**Fig. S3:** Population inference from Structure based on *Basic sample set* and 7329 unlinked SNPs; *K*=3–7, averages of ten runs per *K* (similarity score = 1.0 for each *K*). *Rubus* subsect. *Rubus* (*Suberecti*) includes both North American and European taxa in this order.

**Figure S4**


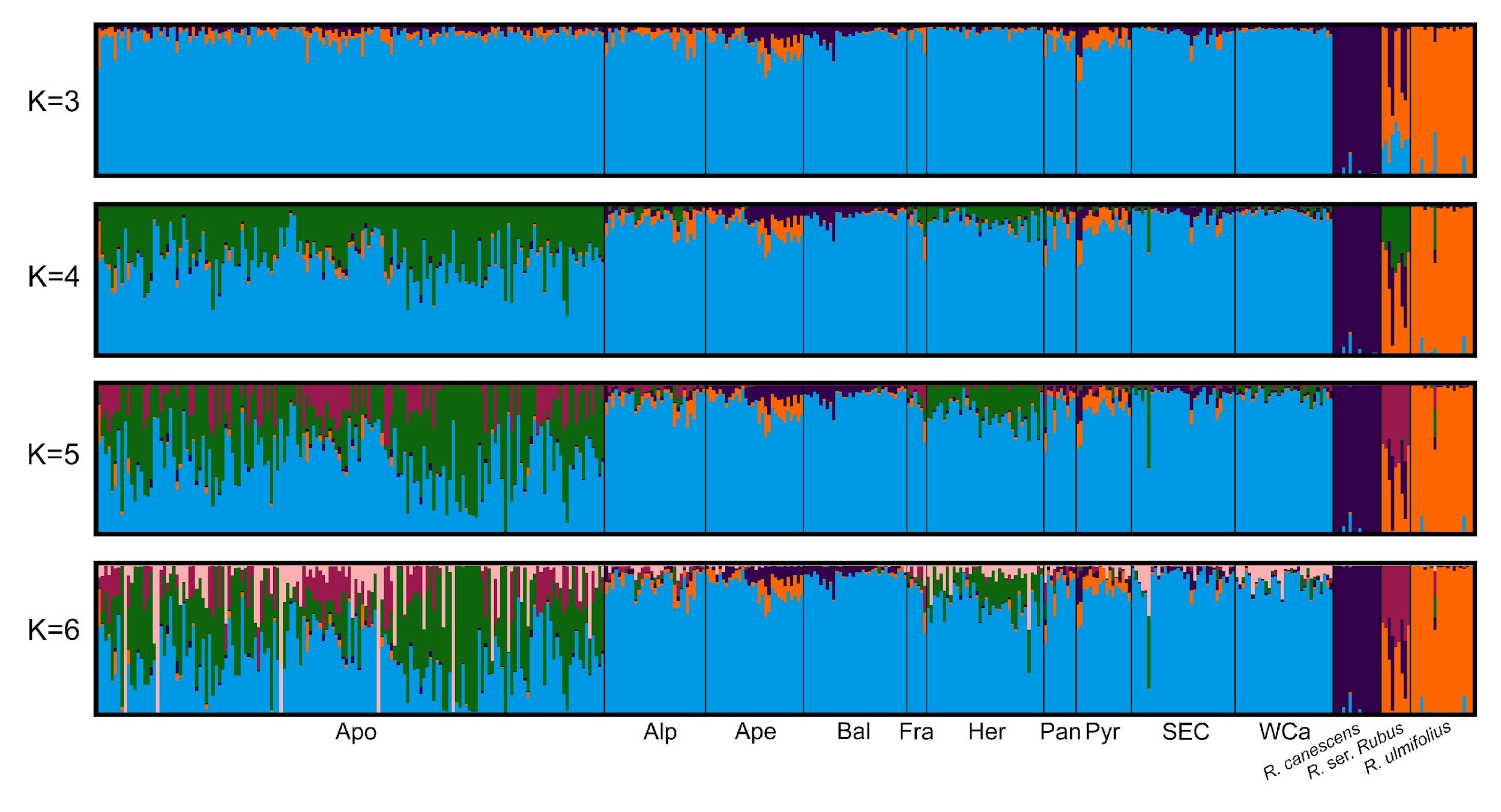


**Fig. S4:** Population inference from Structure based on *European dataset* and 7596 unlinked SNPs; *K*=3–6, averages of ten runs for *K*=3 to 5 (similarity score = 1.0) and six runs (major cluster) for K=6 (similarity score = 0.999).

**Figure S5**





**Fig. S5**: FineRADstructure coancestry matrix visualized as a heatmap based on a complete sample set with 30,328 loci (98,362 variant sites). Populations’ colours correspond to Fig. 1, derived polyploid outgroups other than *R.* subsect. *Rubus* (*Suberecti*) shown with white background.

**Figure S6**


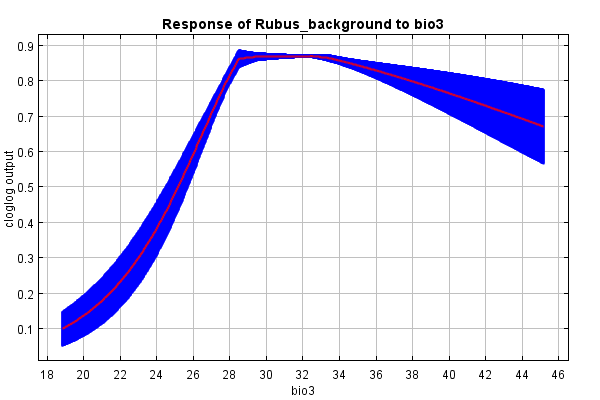

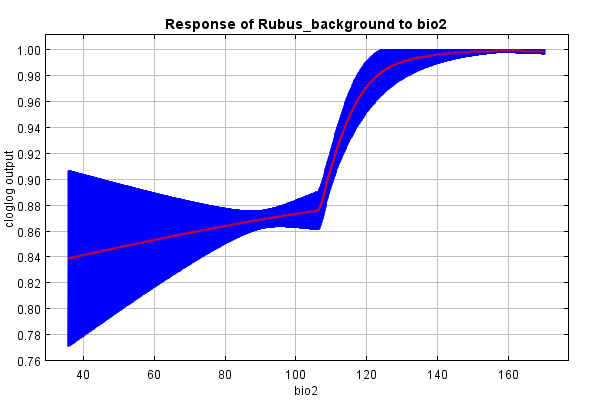

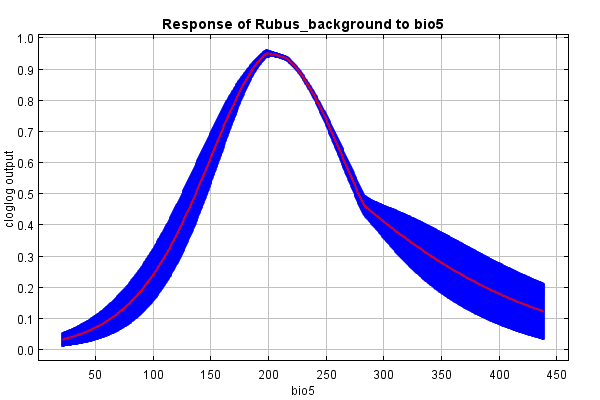

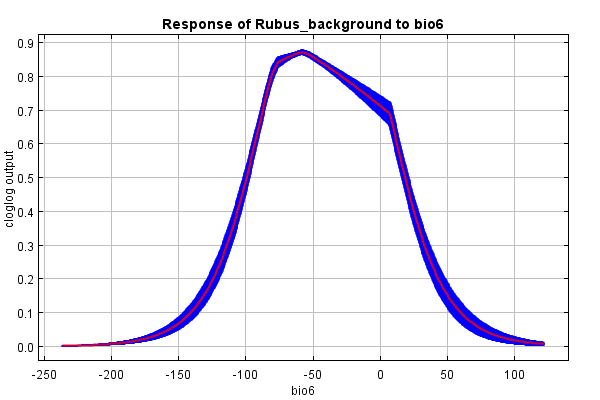

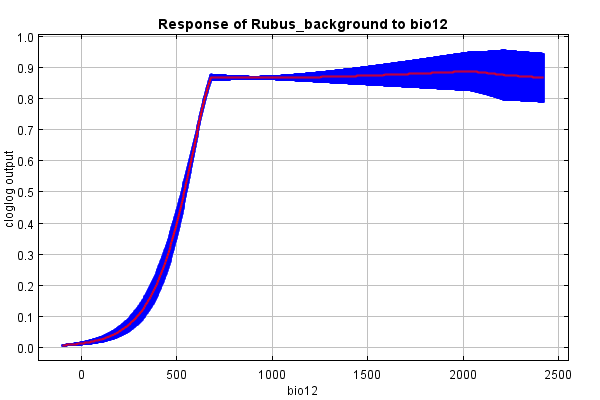

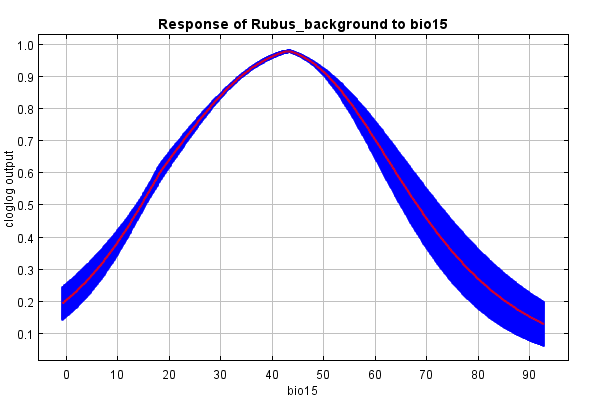


**Fig. 6A:** Response curves showing how each environmental variable affects the Maxent prediction. The curves show how the predicted probability of presence changes as each environmental variable is varied, keeping all other environmental variables at their average sample value (mean+-1 SD from 10 runs).


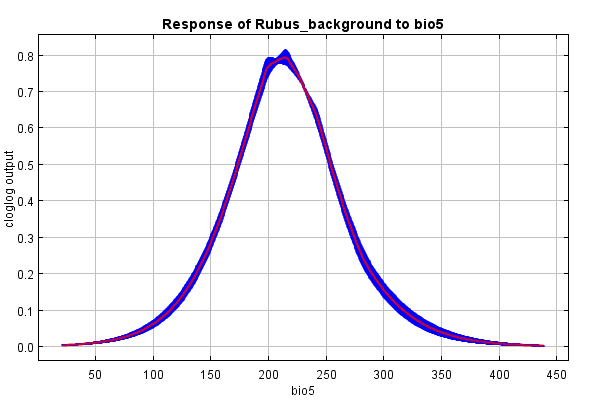

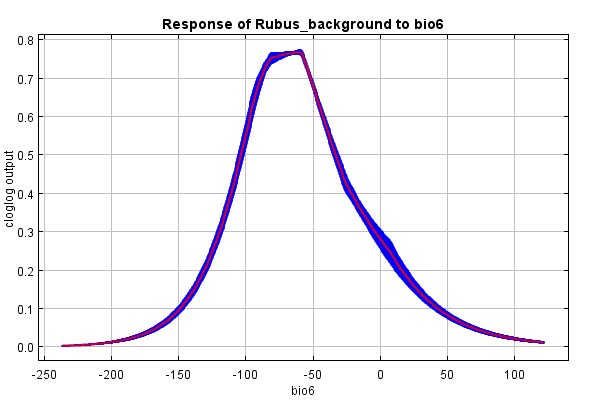

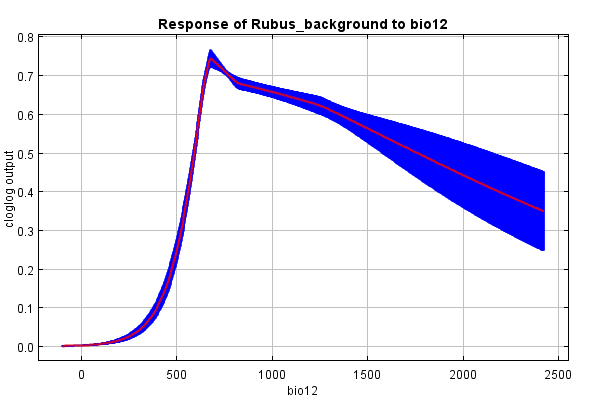

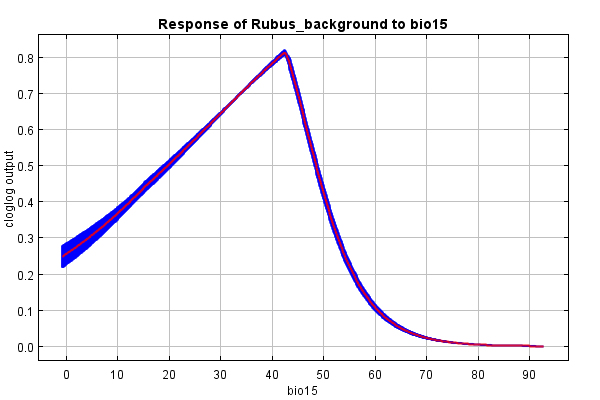

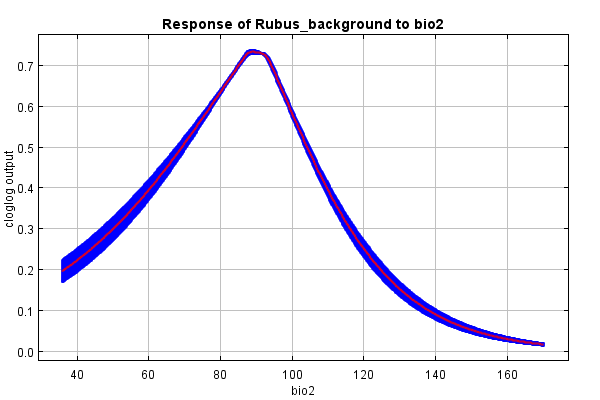

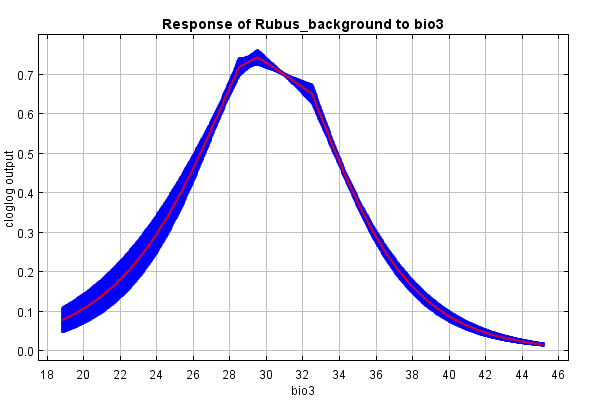


**Fig. 6B:** Marginal response curves; each of the curves represents a different model, namely, a Maxent model created using only the corresponding variable (mean+-1 SD from 10 runs).

**Figure S7**


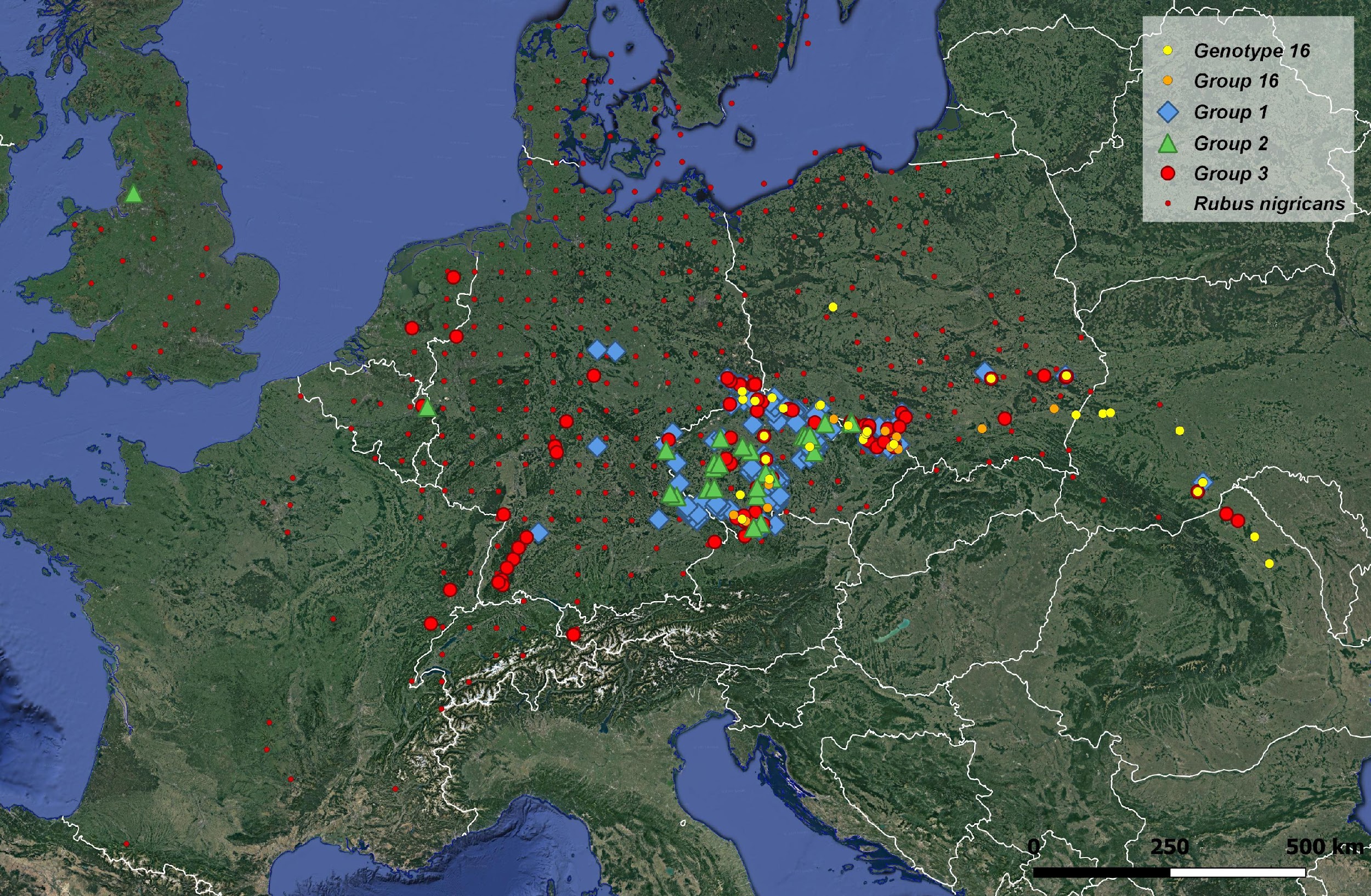


**Fig. S7:** Distribution of apomictic groups as defined by fineRADstructure analysis. All samples used in Sochor et al. (2024) are included according to their genotypic identification; distribution of *Rubus nigricans* belonging to Group 3 is taken from Kurtto et al. (2010; under the name *R. pedemontanus*); Genotype 16 is distinguished here from the rest of Group 16 by a different symbol.
